# L-shaped relationship between dietary vitamin E intake and migraine in adults: a cross-sectional analysis of NHANES 1999–2004

**DOI:** 10.3389/fneur.2025.1582379

**Published:** 2025-06-03

**Authors:** Wangchun Wu, Yanguo Peng, Shuiyu Chen

**Affiliations:** Department of Neurosurgery, The Affiliated Mindong Hospital of Fujian Medical University, Fu’an, China

**Keywords:** dietary, vitamin E intake, migraine, L-shaped, NHANES

## Abstract

**Background:**

Several previous studies have suggested that micronutrients with antioxidant properties are protective factors against migraine. Despite being an important dietary antioxidant, the relationship between dietary vitamin E and migraine has not been extensively studied. This study sought to understand the association between dietary vitamin E intake and the incidence of migraine.

**Methods:**

Data from the National Health and Nutrition Examination Survey (1999–2004) were used in this cross-sectional analysis. To assess the association between dietary vitamin E intake and migraine, we employed logistic regression, restricted cubic spline regression, and stratified analyses.

**Results:**

This study included 10,063 participants who were 20 years old or older, 20.1% (2018/10,063) of whom reported suffering from migraine. Compared with the lowest vitamin E intake T1 (<4.5 mg/day), the adjusted odds ratios (ORs) of dietary vitamin E intake and migraine for T2 (4.5–7.8 mg/day) and T3 (>7.8 mg/day) were 0.85 (95% confidence interval [CI]: 0.74–0.98, *p* = 0.03) and 0.75 (95% confidence interval [CI]: 0.63–0.90, *p* = 0.001), respectively. An L-shaped nonlinear association existed between dietary vitamin E consumption and migraines (*p* = 0.006). For participants who consumed <7.3 mg of vitamin E daily, the OR for migraine was 0.92 (95% CI: 0.874–0.969, *p* = 0.0015). When daily vitamin E intake was ≥7.3 mg/day, the risk of migraine did not continue to decrease with increasing dietary vitamin E consumption (OR, 1.008; 95% CI, 0.984–1.033). The results of the sensitivity analysis suggest that the association between dietary vitamin E intake and migraine remains robust.

**Conclusion:**

In adults, the association between dietary vitamin E intake and migraine is an L-shaped curve (nonlinear, *p* = 0.006), with an inflection point at 7.3 mg/day. It reminds individuals to keep diets balanced.

## 1 Introduction

Migraine, a common chronic neurological condition, is characterized by recurring unilateral headaches that range from moderate to severe in intensity and is accompanied by symptoms such as nausea, phonophobia, and photophobia (1). The Global Burden of Diseases, Injuries, and Risk Factors (GBD) study identified migraine as the second highest cause of neurological disability-adjusted life years (DALYs) worldwide (2). Migraine affects approximately 1 billion individuals worldwide, imposing a significant burden on individuals, families, and society, particularly women aged 15–49 years, who have the highest prevalence (3). The socio-economic cost of migraine in Germany is 100.4 billion euros per year. In the United States, the prevalence of migraines among adults is estimated at 15.9%, affecting approximately 21% of women and 10.7% of men (4). The etiology of migraine continues to be complex, involving multiple factors, and has not yet been fully elucidated. The association between nutrients and migraine has garnered increasing interest in recent years. Several studies have demonstrated that certain nutrients can decrease the frequency of migraines and mitigate their symptoms (5, 6). Consequently, further investigations into additional nutrients potentially linked to migraines could aid in their prevention and treatment.

Vitamin E, an essential nutrient, is involved in numerous biochemical pathways within human cells (7). Vitamin E is recognized as a nutrient with anti-inflammatory (8, 9) and antioxidant (10, 11) properties. Moreover, it is involved in the modulation of signal transduction within neuronal cells (12). Several previous studies have demonstrated that vitamin E supplementation can mitigate both the frequency and severity of migraines (13). However, these studies have concentrated on vitamin E supplementation among individuals with migraines; there is a dearth of research examining the link between dietary vitamin E intake and the incidence of migraines within the general population.

Thus, a cross-sectional study was conducted using data from the National Health and Nutrition Examination Survey (NHANES) to explore the relationship between adult dietary vitamin E intake and the prevalence of migraines. Our research hypothesized that individuals with migraines consume less dietary vitamin E than does the general population. Additionally, this study explored the dose–response relationship between the intake of dietary vitamin E and migraines.

## 2 Materials and methods

### 2.1 Study population

**Figure 1:**
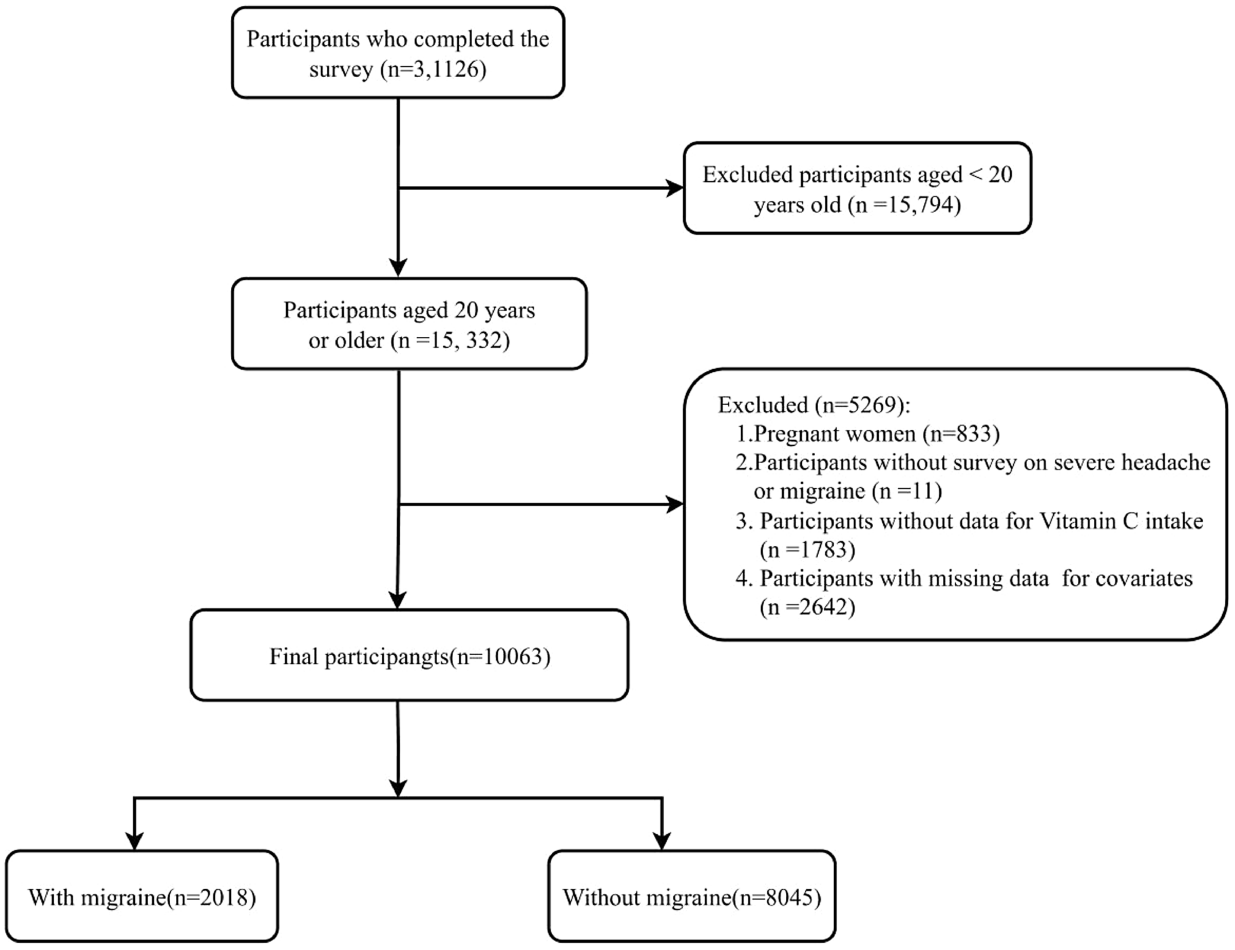
Flow chart of participant selection.

This study utilized data from the NHANES spanning the years 1999–2004. The NHANES, a pivotal program of the National Center for Health Statistics (NCHS), is charged with the assessment of health and nutritional well-being among all age groups within the U. S. population. The interviews and medical examinations were integrated into the survey design. The results of the survey can be utilized to ascertain the incidence of diseases and the associated risk factors. All participants provided written informed consent, and the research protocol was granted approval by the Ethics Review Board of the National Center for Health Statistics (NCHS). All the data are indeed publicly accessible through the official website https://www.cdc.gov/nchs/nhanes/index.htm. A total of 31,126 participants completed interviews from 1999–2004. The participants who were excluded numbered 21,063, comprising those who were pregnant (*n* = 833), aged less than 20 years (*n* = 15,794), with missing migraine data (*n* = 11), incomplete dietary vitamin E intake data (*n* = 1,783), and lacking data on covariates (*n* = 2,642). In conclusion, the cross-sectional analysis included a total of 10,063 participants from the NHANES (1999–2004). The process of participant selection is illustrated in Figure 1.

### 2.2 Assessment of migraine or severe headache

Individuals were classified as migraine sufferers if they confirmed experiencing either a severe headache or a migraine episode within the preceding 3 months, as indicated by their responses on the NHANES Miscellaneous Pain Questionnaire (MPQ). The American Migraine Prevalence and Prevention Study (AMPP) revealed that among the 17.4% of participants who reported severe headaches, 11.8 and 4.6% were classified as having migraines and probable migraines, respectively, on the basis of the International Classification of Headache Disorders-2 (ICHD-2) criteria, whereas 1.0% were classified as having other severe headaches (14). Consequently, it can be inferred that most of the individuals who experienced severe headaches in this study experienced migraines.

### 2.3 Dietary vitamin E intake

All participants in the NHANES underwent 24-h food intake recall to obtain dietary intake information. Dietary intake data collection spanned two methodologies: the NHANES Computer-Assisted Dietary Interview System (CADI) from 1999–2001 and the U. S. Department of Agriculture (USDA) Automated Multiple Passing Method (AMPM) from 2002–2004. The dietary intake data enabled the estimation of total energy, nutrient, and nonnutrient food component intake from food and beverages consumed by participants during the 24-h period preceding the interview. During the 1999–2002 cycle, participants underwent a single dietary recall interview, in contrast to the 2003–2004 cycle, which included two interviews. To maintain consistency with the 1999–2002 data, only the dietary data from the initial interview conducted in the 2003–2004 cycle were considered in this analysis. The NHANES Dietary Interview-Total Nutrient Intakes dataset provided the basis for our analysis of vitamin E consumption. For this study, participants were segmented into tertiles according to their dietary vitamin E levels.

### 2.4 Covariables

Potential covariates were selected for evaluation in accordance with literature and clinical practice (6, 15–17). The analysis included the following variables: demographic factors (age, sex, race and marital status); socioeconomic indicators (family poverty income ratio [PIR] and education level); lifestyle factors (alcohol consumption and smoking status); dietary factors (total cholesterol, protein intake, carbohydrate intake, fat intake, and energy intake); health conditions (hypertension, diabetes, stroke, and coronary heart disease); and C-reactive protein (CRP) levels and body mass index (BMI). The facial classification included non-Hispanic White, non-Hispanic Black, Mexican American, and other. Marital status was divided into married, partnered or living alone. Educational level was divided into < 9, 9–12, or > 12 years. Smoking status was determined by <100 cigarettes (nonsmokers) and ≥100 cigarettes (smokers). Self-reports in a questionnaire were used to ascertain the presence of hypertension, diabetes, stroke, and coronary heart disease (CHD). Alcohol consumption was quantified on the basis of the frequency of alcohol intake over the preceding 12 months.

### 2.5 Statistical analyses

This research represents a secondary analysis of publicly accessible datasets. Variable normality was assessed via histogram plots, Q–Q plots, and Kolmogorov–Smirnov tests. Normally distributed continuous variables are presented as the means ± standard deviations (SD), whereas skewed continuous variables are presented as medians with interquartile ranges (IQR). Categorical variables are displayed as frequencies and percentages. To assess group differences across variable types, one-way analyses of variance (ANOVA) were employed for normally distributed continuous variables, the Kruskal–Wallis test was used for skewed continuous variables, and the chi–square test was utilized for categorical variables. Dietary vitamin E was classified into tertiles. To ascertain the relationships between different tertiles of vitamin E consumption and migraine, logistic regression models were used to calculate the odds ratios (OR) and 95% confidence interval (CI). Three models were constructed for the analysis.

Furthermore, with all covariates adjusted, we employed a restricted cubic spline (RCS) model to create smooth curves to investigate potential nonlinear dose–response relationships between dietary vitamin E intake and migraine. In instances where a nonlinear relationship was detected, a two-piecewise regression model was used to determine the threshold effect of dietary vitamin E intake on migraine. Likelihood ratio tests and the bootstrap resampling method were used to find inflection points. Interaction and stratified analyses were performed across subgroups. Multivariate logistic regression was used to evaluate heterogeneity within these subgroups, and likelihood ratio tests pinpointed interactions between subgroup variables and vitamin E intake. To ascertain the reliability of our findings, sensitivity analyses were conducted, excluding participants whose energy intake values were less than 500 kcal per day or greater than 5,000 kcal per day. All the statistical analyses were performed via R Statistical Software (Version 4.2.2, The R Foundation) and the Free Statistical Analysis Platform (Version 2.0, Beijing, China). Statistical significance was defined as a two-tailed *p* value < 0.05.

## 3 Results

### 3.1 Basic characteristics of the participants

Table 1 presents the baseline characteristics of the 10,063 participants, stratified by vitamin E intake tertile. The mean age was 50.3 (18.5) years, and 5,084 (50.5%) participants were male. A total of 2,018 individuals (20.1%) reported suffering from migraine. Individuals with greater vitamin E intake, often younger, male, non-Hispanic White, well educated, married or cohabiting, and nonsmoking, presented lower rates of hypertension, diabetes, stroke, and coronary heart disease; higher energy, carbohydrate, protein, and fat consumption; a higher family poverty–income ratio; and reduced serum C-reactive protein levels. The univariate analysis revealed statistically significant associations between migraine and the following factors: age, sex, race, CHD, total cholesterol, protein, carbohydrate, family PIR, and BMI (Table 2).

**Table 1:** Characteristics of population by dietary vitamin E intake levels.

Characteristics	Vitamin E intake, mg/day				
	Total	T1	T2	T3	*p*-value
		(<4.5)	4.5–7.8	>7.8	
NO.	10,063	3,346	3,358	3,359	
Age (year), Mean ± SD	50.3 ± 18.5	52.8 ± 18.9	50.2 ± 18.5	48.1 ± 17.6	< 0.001
Sex, *n* (%)					< 0.001
Male	5,084 (50.5)	1,407 (42.1)	1,637 (48.7)	2040 (60.7)	
Female	4,979 (49.5)	1939 (57.9)	1721 (51.3)	1,319 (39.3)	
Marital status, *n* (%)					< 0.001
Married or Living with a partner	6,303 (62.6)	1929 (57.7)	2,138 (63.7)	2,236 (66.6)	
Living alone	3,760 (37.4)	1,417 (42.3)	1,220 (36.3)	1,123 (33.4)	
Race, *n* (%)					< 0.001
Non-Hispanic White	5,317 (52.8)	1,618 (48.4)	1795 (53.5)	1904 (56.7)	
Non-Hispanic Black	1874 (18.6)	734 (21.9)	599 (17.8)	541 (16.1)	
Mexican American	2,187 (21.7)	760 (22.7)	733 (21.8)	694 (20.7)	
Others	685 (6.8)	234 (7)	231 (6.9)	220 (6.5)	
Education level (years), *n* (%)					< 0.001
<9	1,462 (14.5)	670 (20)	442 (13.2)	350 (10.4)	
9–12	4,008 (39.8)	1,441 (43.1)	1,307 (38.9)	1,260 (37.5)	
>12	4,593 (45.6)	1,235 (36.9)	1,609 (47.9)	1749 (52.1)	
Smoking, *n* (%)					0.089
No	5,076 (50.4)	1,644 (49.1)	1740 (51.8)	1,692 (50.4)	
Yes	4,987 (49.6)	1702 (50.9)	1,618 (48.2)	1,667 (49.6)	
Hypertension, *n* (%)					< 0.001
No	6,806 (67.6)	2,132 (63.7)	2,317 (69)	2,357 (70.2)	
Yes	3,257 (32.4)	1,214 (36.3)	1,041 (31)	1,002 (29.8)	
Diabetes, *n* (%)					< 0.001
No	9,044 (89.9)	2,927 (87.5)	3,034 (90.4)	3,083 (91.8)	
Yes	1,019 (10.1)	419 (12.5)	324 (9.6)	276 (8.2)	
Stroke, *n* (%)					< 0.001
No	9,742 (96.8)	3,203 (95.7)	3,253 (96.9)	3,286 (97.8)	
Yes	321 (3.2)	143 (4.3)	105 (3.1)	73 (2.2)	
Coronary heart disease, *n* (%)					0.006
No	9,589 (95.3)	3,161 (94.5)	3,199 (95.3)	3,229 (96.1)	
Yes	474 (4.7)	185 (5.5)	159 (4.7)	130 (3.9)	
Alcohol, Median (IQR)	2.0 (1.0, 3.0)	2.0 (0.0, 3.0)	2.0 (1.0, 3.8)	2.0 (1.0, 4.0)	< 0.001
C-reactive protein (mg/dl), Median (IQR)	0.2 (0.1, 0.5)	0.3 (0.1, 0.6)	0.2 (0.1, 0.5)	0.2 (0.1, 0.4)	< 0.001
Total cholesterol (mmol/L), Mean ± SD	5.2 ± 1.1	5.3 ± 1.1	5.3 ± 1.1	5.2 ± 1.1	0.002
Protein intake (g/day), Mean ± SD	79.8 ± 42.0	56.8 ± 29.1	78.3 ± 32.3	104.2 ± 47.8	< 0.001
Carbohydrate intake (g/day), Mean ± SD	262.9 ± 134.7	188.5 ± 91.2	261.4 ± 109.3	338.6 ± 151.2	< 0.001
Fat (g/day), Mean ± SD	79.1 ± 46.3	48.9 ± 25.1	76.0 ± 31.3	112.3 ± 52.8	< 0.001
Energy (kcal/day), Mean ± SD	2125.7 ± 1029.6	1468.7 ± 647.1	2080.3 ± 739.7	2825.4 ± 1136.0	< 0.001
BMI (kg/m2), Mean ± SD	28.3 ± 6.2	28.4 ± 6.2	28.2 ± 6.1	28.3 ± 6.2	0.441
Family PIR, Mean ± SD	2.7 ± 1.6	2.4 ± 1.6	2.7 ± 1.6	2.9 ± 1.6	< 0.001

PIR, poverty income ratio; BMI, body mass index. T1-T3, tertile based on dietary Vitamin E intake. Categorical variables: proportions (%); Continuous variables: means ± SD (standard deviation) or medians (interquartile range, IQR).

**Table 2:** Association between covariates and migraine risk.

Variable	OR (95% CI)	*p*_value
Age (years)	0.98 (0.97 ~ 0.98)	<0.001
Sex, *n* (%)		
Male	1 (reference)	
Female	2.16 (1.95 ~ 2.39)	<0.001
Marital status, *n* (%)		
Married or Living with a partner	1 (reference)	
Living alone	1.1 (0.99 ~ 1.22)	0.064
Race, *n* (%)		
Non-Hispanic White	1 (reference)	
Non-Hispanic Black	1.28 (1.12 ~ 1.45)	<0.001
Mexican American	1.22 (1.08 ~ 1.38)	0.001
Others	1.34 (1.11 ~ 1.62)	0.003
Education level (years), *n* (%)		
<9	1 (reference)	
9–12	1.15 (0.99 ~ 1.33)	0.07
>12	0.9 (0.77 ~ 1.04)	0.156
Smoking, *n* (%)		
No	1 (reference)	
Yes	0.95 (0.86 ~ 1.05)	0.294
Hypertension, *n* (%)		
No	1 (reference)	
Yes	0.97 (0.88 ~ 1.08)	0.626
Diabetes, *n* (%)		
No	1 (reference)	
Yes	0.92 (0.78 ~ 1.09)	0.349
Stroke, *n* (%)		
No	1 (reference)	
Yes	1.16 (0.89 ~ 1.51)	0.28
Coronary heart disease, *n* (%)		
No	1 (reference)	
Yes	0.62 (0.48–0.81)	<0.001
Alcohol	1 (1.00–1.00)	0.54
C-reactive protein (mg/dl)	1.04 (1.00–1.09)	0.076
Total cholesterol (mmol/L)	0.94 (0.9–0.99)	0.013
Protein intake (g/day)	1.00 (1.00–1.00)	0.02
Carbohydrate intake (g/day)	1.00 (1.00–1.00)	0.026
Fat (g/day)	1.00 (1.00–1.00)	0.652
Energy (kcal/day)	1.00 (1.00–1.00)	0.679

CI, confidence interval; OR, odds ratio; PIR, poverty income ratio; BMI, body mass index.

### 3.2 Correlations between vitamin E intake and migraine

**Figure 2:**
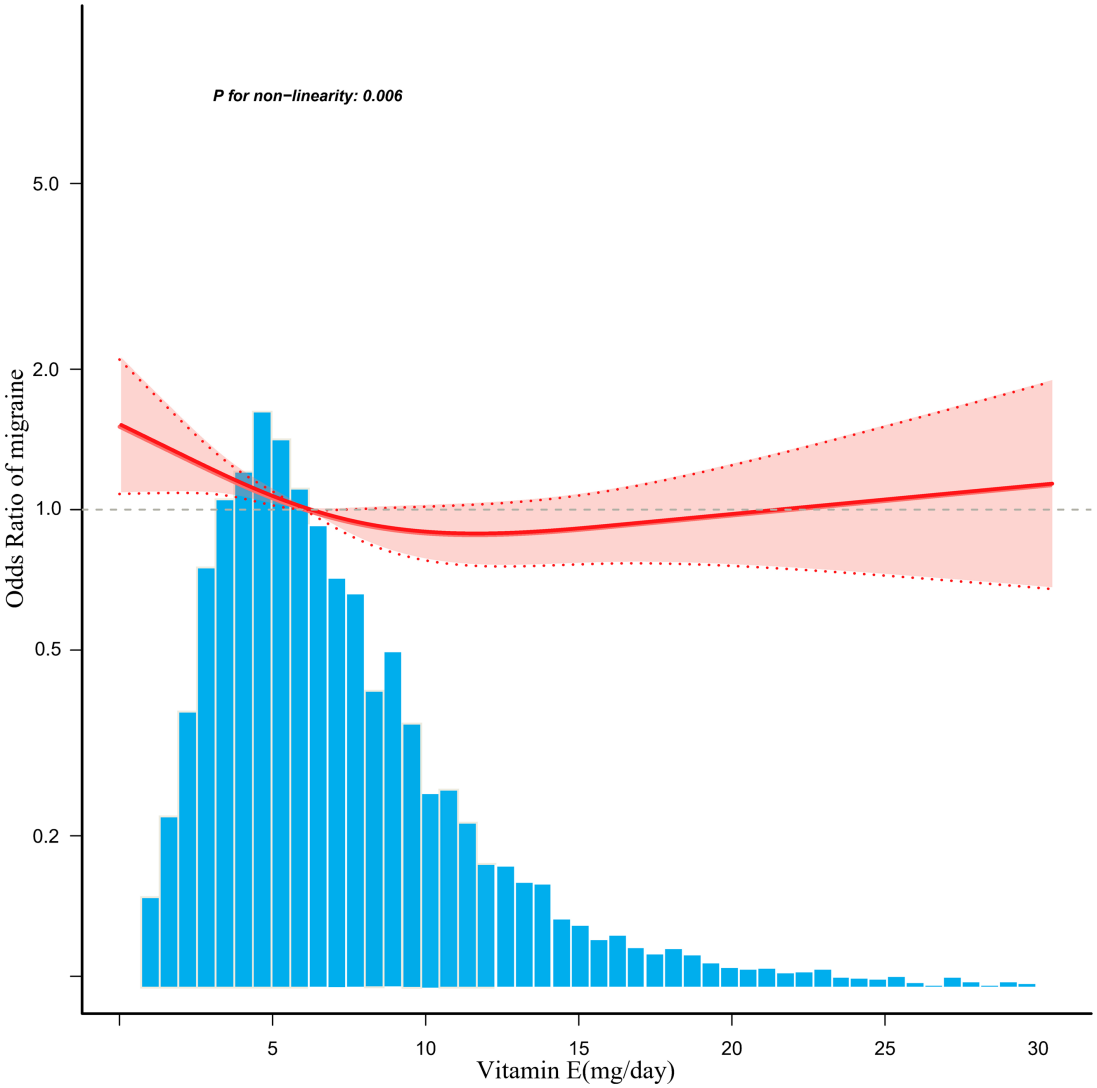
Associations between dietary vitamin E intake and the migraine odds ratio. The solid and dashed lines represent the predicted values and 95% confidence intervals, respectively. They were adjusted for age, sex, marital status, race, education level, total cholesterol, protein intake, carbohydrate intake, fat, energy, family poverty income ratio, C-reactive protein, body mass index, alcohol, smoking, hypertension, diabetes, stroke, and coronary heart disease. Only 99% of the data are displayed.

A significant inverse association was identified between dietary vitamin E intake and migraine after adjusting for potential confounding variables when dietary vitamin E intake was analyzed using tertiles (T). Compared with the lowest vitamin E intake T1 (<4.5 mg/day), the adjusted odds ratios of dietary vitamin E intake and migraine for T2 (4.5–7.8 mg/day) and T3 (>7.8 mg/day) were 0.85 (95% CI: 0.74–0.98, *p* = 0.03) and 0.75 (95% CI: 0.63–0.90, *p* = 0.001), respectively (Table 3). An L-shaped nonlinear association existed between dietary vitamin E consumption and migraines (*p* = 0.006) in the RCS (Figure 2). In the threshold analysis, for participants who consumed <7.3 mg of vitamin E daily, the OR for migraine was 0.92 (95% CI: 0.874–0.969, *p* = 0.0015) (Table 4). This finding indicates that each 1 mg increase in daily dietary vitamin E intake is associated with an 8% reduction in the risk of migraine. However, when daily vitamin E intake was ≥7.3 mg/day, the risk of migraine did not continue to decrease with increasing dietary vitamin E consumption (OR, 1.008; 95% CI, 0.984–1.033) (Table 4).

**Table 3:** Association between dietary vitamin E intake and migraine.

Exposure	No.	Crude model		Model 1		Model 2		Model 3	
		OR (95% CI)	*p*-value	OR (95% CI)	*p*-value	OR (95% CI)	*p*-value	OR (95% CI)	*p*-value
Vitamin E (mg/d)									
T1 (<4.5)	3,346	1 (Ref)		1 (Ref)		1 (Ref)		1 (Ref)	
T2 (4.5–7.8)	3,358	0.88 (0.78 ~ 0.99)	0.035	0.9 (0.8 ~ 1.02)	0.087	0.83 (0.71 ~ 0.95)	0.009	0.85 (0.74 ~ 0.98)	0.03
T3 (>7.8)	3,359	0.78 (0.7 ~ 0.88)	<0.001	0.85 (0.75 ~ 0.96)	0.012	0.72 (0.61 ~ 0.86)	<0.001	0.75 (0.63 ~ 0.9)	0.001
Trend.test	10,063	0.89 (0.83 ~ 0.94)	<0.001	0.92 (0.86 ~ 0.98)	0.012	0.85 (0.78 ~ 0.92)	<0.001	0.87 (0.8 ~ 0.95)	0.001

Model 1 was adjusted for age, sex, marital status, race and education level.

Model 2 was adjusted for Model 1 + alcohol, smoking, C-reactive protein, total cholesterol, protein intake, carbohydrate intake, fat, energy.

Model 3 was adjusted for model 2 + BMI, family PIR, hypertension, diabetes, stroke, and coronary heart disease.

BMI, body mass index; PIR, poverty income ratio; CI, confidence interval; OR, odds ratio; T, tertile.

**Table 4:** Analysis of the threshold effect of vitamin E intake on migraine risk.

Vitamin E (mg/d)	Adjusted model	
	OR (95% CI)	*p*-value
<7.3	0.92 (0.874 ~ 0.969)	0.0015
≥7.3	1.008 (0.984 ~ 1.033)	0.5066
Likelihood ratio test		0.003

OR, odds ratio; CI, confidence interval. Adjusted for age, sex, marital status, race, education level, total cholesterol, protein intake, carbohydrate intake, fat, Energy, family poverty income ratio, C-reactive protein, body mass index, alcohol, smoking, hypertension, diabetes, stroke, and coronary heart disease. Only 99% of the data is displayed.

### 3.3 Stratified and interaction analyses

To evaluate the potential impact of the relationship between dietary vitamin E intake and migraine, stratified analyses were conducted. No statistically significant interactions were identified in any of the subgroups stratified by age, sex, marital status, education level, BMI, stroke status, diabetes status, or hypertension status (Supplementary Figure S1).

### 3.4 Sensitivity analysis

Following the exclusion of individuals with abnormal energy intake, the correlation between dietary vitamin E intake and migraine remained consistent among the 9,806 remaining participants. Compared with the lowest vitamin E intake T1 (<4.5 mg/day), the adjusted odds ratios of dietary vitamin E intake and migraine for T2 (4.5–7.7 mg/day) and T3 (>7.7 mg/day) were 0.88 (95% CI: 0.76–1.02, *p* = 0.096) and 0.77 (95% CI: 0.65–0.92, *p* = 0.005), respectively (Supplementary Table S1).

## 4 Discussion

In a cross-sectional analysis of 10,063 U. S. adults, an L-shaped association between dietary vitamin E intake and migraine was identified, with an inflection point at approximately 7.3 mg/day. Stratified and sensitivity analyses both demonstrated that the association between dietary vitamin E intake and migraine was robust.

The influence of vitamin E on migraine has been documented in only a few studies. Visser et al. administered a combination of antioxidants with vitamin E to 35 individuals with migraine. Notably, Visser et al. reported significant decreases in monthly migraine frequency, duration of migraine attacks, headache severity scores, and the use of acute headache medications among participants, implying that vitamin E could serve as a potential adjunct therapy for migraine (13). In a placebo-controlled, double-blind trial conducted by Saeideh Ziaei et al., 72 women experiencing menstrual migraine reported relief after taking vitamin E capsules (400 IU) daily for 5 days (18). A literature review conducted by Shaik MM et al. suggested that vitamin E may confer benefits for individuals experiencing migraines (19). Chayasirisobhon et al. used antioxidant therapy, including vitamin E, to reduce the frequency and severity of migraine, suggesting that vitamin E might play a positive role in migraine. (20). To date, no research has investigated the potential association between adult dietary vitamin E consumption and migraine in the general population. The NHANES dataset offers a unique opportunity to evaluate the potential association between dietary vitamin E and migraine, including the dose–response relationship.

The association between dietary vitamin E intake and migraine follows an L-shaped pattern. Specifically, for individuals with a daily intake below the threshold of 7.3 mg, an increase in vitamin E consumption is associated with a lower risk of migraines. However, this association is not observed in individuals with an intake above 7.3 mg/day. The primary dietary sources of vitamin E include seeds, certain fruits, leafy vegetables, vegetable oils, and nuts (21). Our findings, when integrated with dietary considerations, suggest that a balanced diet might contribute to migraine prevention. For example, the Mediterranean diet may be rich in vitamin E due to its abundance of legumes, fruits, vegetables, olive oil, and nuts (22). A study conducted by Arab et al. demonstrated that adherence to a Mediterranean dietary pattern was linked to reduced migraine frequency, duration, and severity (23). A study by Hande Bakırhan et al. indicated that a high-quality dietary pattern may alleviate the symptoms of individuals with migraine (24).

Although the specific biological mechanisms explaining the inverse association between vitamin E and migraine remain to be explored, our findings are biologically plausible and supported by the available evidence. First, some studies have shown that oxidative stress may be involved in causing migraine (25, 26). Free radicals, which are highly reactive chemical species, can induce cellular and tissue damage when their levels become excessive. Overproduction of free radicals that exceed the body’s antioxidant defense mechanisms results in disruption of the redox equilibrium, culminating in a state of oxidative stress (26, 27). Vitamin E mitigates oxidative stress through the scavenging of free radicals and potentiates the effects of other antioxidants, thereby increasing the overall antioxidant capacity (10). Second, the inflammatory response also plays an important role in the pathogenesis of migraine. Several studies have demonstrated an association between migraines and the release of prostaglandins, a key inflammatory mediator (28). Vitamin E inhibits prostaglandin synthesis by acting on phospholipase A2 and cyclooxygenase, thereby reducing the inflammatory response (29). Third, vitamin E can affect neuroinflammation and oxidative stress through multiple signaling pathways (30). Fourth, vitamin E prevents oxidative damage to vascular endothelial cells, maintains normal vascular function, and reduces vasodilation during migraine attacks (31).

The present study has certain limitations. First, the study sample was restricted to U. S. adults aged 20 years or older during the 1999–2004 NHANES period; hence, additional research is needed to ascertain the applicability of these results to different populations. Second, notwithstanding the application of regression modeling, stratification analysis, and sensitivity analysis, the potential impact of unmeasured or unknown confounders on the study’s results cannot be completely discounted. Third, data on severe headache or migraine were obtained from self-report questionnaires, which are not based on the ICHD; however, the prevalence of migraine or severe headache reported in our sample was consistent with existing population estimates, as supported by previous research (14, 32). Fourth, dietary vitamin E intake data were derived from 24-h recall, potentially introducing recall bias. Finally, the cross-sectional design of this study precludes the establishment of a causal link between vitamin E intake and migraine; therefore, future prospective cohort studies are essential to confirm this association.

## 5 Conclusion

In conclusion, our results show that the association between dietary vitamin E intake and migraine in U. S. adults is L shaped, with an inflection point at approximately 7.3 mg/day. For the prevention and treatment of migraine, vitamin E may offer new strategies. It also reminds individuals to keep diets balanced.

## Data Availability

Publicly available datasets were analyzed in this study. This data can be found at: https://www.cdc.gov/nchs/nhanes/?CDC_AAref_Val=https://www.cdc.gov/nchs/nhanes/index.htm.

## Glossary

NHANES: National Health and Nutrition Examination Survey
GBD: Global Burden of Diseases
T: Tertiles
OR: Odds Ratios
DALYs: Disability-Adjusted Life Years
NCHS: National Center for Health Statistics
MPQ: Miscellaneous Pain Questionnaire
AMPP: American Migraine Prevalence and Prevention Study
ICHD-2: International Classification of Headache Disorders-2
CADI: Computer-Assisted Dietary Interview System
USDA: U. S. Department of Agriculture
AMPM: Automated Multiple Passing Method
PIR: Poverty Income Ratio
CRP: C-Reactive Protein
BMI: Body Mass Index
CHD: Coronary Heart Disease
SD: Standard Deviation
IQR: Interquartile Ranges
ANOVA: Analyses of Variance
CI: Confidence Interval
RCS: Restricted Cubic Spline
