# Supplementary material for: L-shaped relationship between dietary vitamin E intake and migraine in adults: a cross-sectional analysis of NHANES 1999–2004
Source: Front Neurol. 2025 Jun 3;16:1582379. doi: 10.3389/fneur.2025.1582379 (PMC12172504; doi:10.3389/fneur.2025.1582379)
Supplement: Supplementary file 1 [file Supplementary_file_1.docx]

Supplementary Table S1 Association between dietary vitamin E intake and migraine.

| Exposure |  | Crude Model | | Model 1 | | Model 2 | | Model 3 | |
| --- | --- | --- | --- | --- | --- | --- | --- | --- | --- |
|  | No. | OR (95%CI) | P-value | OR (95%CI) | P-value | OR (95%CI) | P-value | OR (95%CI) | P-value |
| Vitamin E(mg/d) |  |  |  |  |  |  |  |  |  |
| T1 (<4.5) | 3269 | 1(Ref) |  | 1(Ref) |  | 1(Ref) |  | 1(Ref) |  |
| T2 (4.5-7.7) | 3262 | 0.91 (0.8~1.02) | 0.103 | 0.92 (0.82~1.05) | 0.216 | 0.86 (0.74~1) | 0.043 | 0.88 (0.76~1.02) | 0.096 |
| T3 (>7.7) | 3275 | 0.78 (0.69~0.89) | <0.001 | 0.85 (0.75~0.97) | 0.012 | 0.74 (0.62~0.89) | 0.001 | 0.77 (0.65~0.92) | 0.005 |
| Trend.test | 9806 |  | <0.001 |  | 0.012 |  | 0.001 |  | 0.005 |

Model 1 was adjusted for age, gender,marital status, race and education level.

Model 2 was adjusted for Model 1 + alcohol,smoking,C-reactive protein,total cholesterol,protein intake,carbohydrate intake,fat,

energy.

Model 3 was adjusted for model 2 + BMI,family PIR,hypertension, diabetes, stroke, and coronary heart disease.

Abbreviations: BMI, body mass index;PIR,the ratio of income to poverty; CI, confidence interval; OR, odds ratio; T, tertile


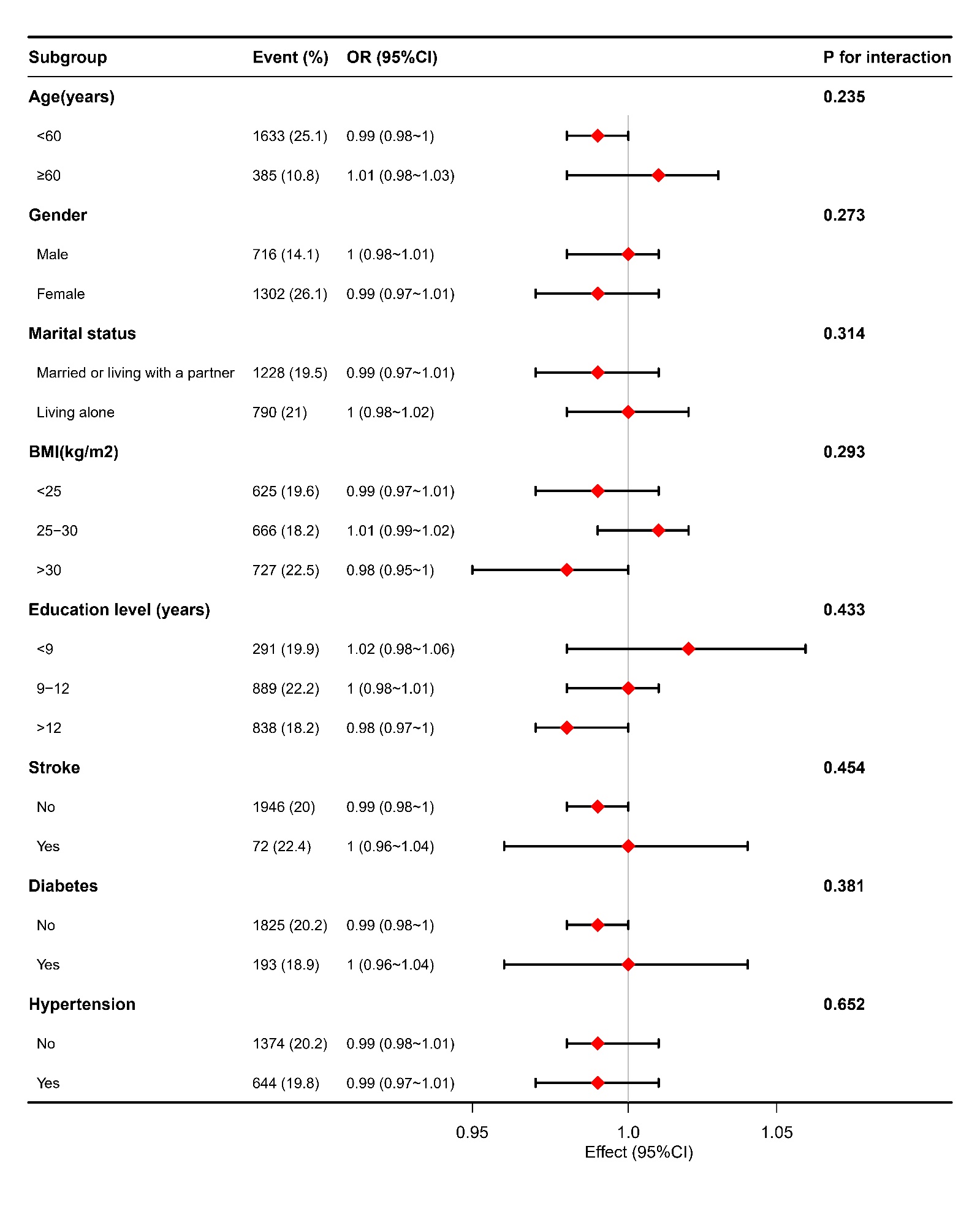
 Supplementary Figure S1. Stratified analyses of the association between dietary vitamin E intake and migraine. With the exception of itself, each stratification variable was adjusted for all other variables (age, sex, marital status, race and education level, total cholesterol, protein intake, carbohydrate intake, fat, energy, family poverty income ratio, C-reactive protein, body mass index, alcohol, smoking, hypertension, diabetes, stroke, and coronary heart disease).
